# Physicochemical Characterization Cascade of Nanoadjuvant–Antigen Systems for Improving Vaccines

**DOI:** 10.3390/vaccines9060544

**Published:** 2021-05-21

**Authors:** Giuditta Guerrini, Antonio Vivi, Sabrina Gioria, Jessica Ponti, Davide Magrì, Arnd Hoeveler, Donata Medaglini, Luigi Calzolai

**Affiliations:** 1 Laboratory of Molecular Microbiology and Biotechnology, Department of Medical Biotechnologies, University of Siena, 53100 Siena, Italy; guerrini12@student.unisi.it (G.G.); antonio.vivi@unisi.it (A.V.); donata.medaglini@unisi.it (D.M.); 2 European Commission, Joint Research Centre (JRC), 21027 Ispra, Italy; Sabrina.GIORIA@ec.europa.eu (S.G.); Jessica.PONTI@ec.europa.eu (J.P.); Davide.MAGRI@ec.europa.eu (D.M.); Arnd.Hoeveler@ec.europa.eu (A.H.)

**Keywords:** adjuvants, antigens, characterization, vaccines

## Abstract

Adjuvants have been used for decades to enhance the immune response to vaccines, in particular for the subunit-based adjuvants. Physicochemical properties of the adjuvant-protein antigen complexes, such as size, morphology, protein structure and binding, influence the overall efficacy and safety of the vaccine. Here we show how to perform an accurate physicochemical characterization of the nanoaluminum–ovalbumin complex. Using a combination of existing techniques, we developed a multi-staged characterization strategy based on measurements of increased complexity. This characterization cascade has the advantage of being very flexible and easily adaptable to any adjuvant-protein antigen combinations. It will contribute to control the quality of antigen–adjuvant complexes and immunological outcomes, ultimately leading to improved vaccines.

## 1. Introduction

Adjuvants are used in vaccines to enhance the immunogenicity of the antigen and are particularly important in the case of subunit vaccines where the recombinant protein antigens are poorly immunogenic and adjuvants are added as nonspecific immunostimulants to increase the efficacy of the vaccine formulation [1]. Among them, aluminum-based adjuvants have been used since 1932 in licensed human vaccines and, for nearly seven decades, they have been the only adjuvants authorized for human vaccines [2]. Their long-term success is mainly due to their safety profile, relatively low cost, and capacity to bind a large variety of subunit antigens [3]. The two types of aluminum adjuvants normally used in licensed vaccines are aluminum hydroxide (AH) and aluminum phosphate (AP), which can bind negatively or positively charged antigens. In addition, the surface charge of these adjuvants is pH-dependent, and their capacity of binding protein antigens can be fine-tuned by changing either the pH or the ionic composition of the buffer in which the adjuvants and/or the antigens are used.

Remarkably, despite the significant improvement in the formulation of new vaccines, aluminum-based vaccines are still used due to their ability to cover safety requirements with simple and inexpensive formulations. Aluminum-based vaccines have also been developed against COVID-19. For example, Adimmune is developing a vaccine containing the recombinant receptor-binding domain (RBD) of SARS-CoV-2 spike protein with aluminum adjuvant, which is now in clinical trials [4,5].

Several studies demonstrated the mechanism of immunopotentiation carried out by aluminum, from the enhanced recruitment of antigen-presenting cells (APC) at the injection site, to the improved antigen uptake and maturation of APC [6].

According to the World Health Organization (WHO), the mechanism of action of aluminum-based adjuvants is still not completely understood; according to the World Health Organization (WHO), at least 80% of the antigen should be bound to the adjuvant itself. This allows the antigen to remain localized at the injection site for prolonged time, avoiding its spreading and dispersion in the interstitial fluids [7].

In addition to the physicochemical properties of the adjuvant, a key role is played by the nature of the interaction between adjuvant and antigen. To date, a comprehensive understanding of the physicochemical properties of the aluminum–antigen complex is lacking. Normally, electrostatic attraction, hydrophilic interaction, and ligand exchange occurs between antigen and adjuvant, rather than a covalent bond [8].

The stability of the recombinant antigen is a key aspect during the development of subunit vaccines. In fact, the conformational stability of antigens has a strong impact on immunogenicity and immune polarization. For example, proteolytic degradation of the protein is required before antigenic peptides are loaded on significant histocompatibility complex receptors. The structural stability of the protein influence the kinetics of the degradation (and ultimately its immunogenicity [9]), while the absorption of proteins on the adjuvant surface may also affect the protein structure and stability [10,11]. Thus, when developing “new subunit vaccines”, it is important to characterize the whole protein–adjuvant complex [12,13].

However, measuring the protein structure and stability of antigen-protein complexes is particularly challenging due to the heterogeneous nature of the system and the low concentrations. To date, only few studies are available in the literature; in particular, circular dichroism (CD) has provided information on the changes in structure and stability of proteins bound to different types of nanoparticles [14,15].

At nanoscale, the antigens binding capacity per adjuvant mass is higher than that of traditional aluminum hydroxide-based adjuvants. This is linked to the smaller particle size, larger specific surface area, higher surface reactivity, and stronger adsorption capacity [16]. In addition, the use of nano-formulations is attracting increased interest, not only as nanomedicines [17], but also as nanovaccines [18]. For example, lipid nanoparticles carriers are used as nano-adjuvants in mRNA-based vaccines [19] against COVID-19. In fact, there is increasing evidence that adjuvants with particles in the size range of 80–150 nm have a more potent adjuvant activity than large microparticles [20]. In particular, nanoaluminum adjuvants, such as aluminum hydroxide nanoparticles (AH-NP), have been shown to significantly mitigate excessive inflammatory reactions (e.g., subcutaneous granuloma) at the injection site [16] and to elicit a Th1 response [21], compared to conventional aluminum hydroxide-based adjuvant. Mitigation of the inflammatory reaction could be important for clinical conditions characterized by defective immune system regulation, where particular attention on adjuvant administration should be taken [22,23].

Aiming to develop a robust characterization for nanoaluminum-subunit vaccines, we have selected nano-AH-ovalbumin as a model system. Ovalbumin (OVA) is a “classical” reference protein for immunization experiments, especially in mice [24,25,26]. It is a phosphorylated globular glycoprotein with 385 amino acids, molecular weight (MW) of 42.7 kDa, predicted isoelectric point (IEP) of 4.5 and a secondary structure made by 30% α-helix and 32% β-sheet by X-ray diffraction [27,28].

In this article, we propose a characterization cascade based on existing techniques for the accurate measurement of the critical physicochemical properties of nanoaluminum adjuvants and nanoadjuvant–antigen systems. This integrated experimental approach is general and is applicable to different adjuvant–antigen combinations. Its use will give a very accurate characterization of the adjuvant–antigen complex, leading to the faster development of a wide variety of much-needed vaccines.

## 2. Materials and Methods

### 2.1. Materials

Alhydrogel^®^ (Oxyhydroxy aluminum) was supplied by Brenntag Biosector (Essen, Germany). OVA from chicken egg and human serum albumin (HSA) were supplied by Sigma Aldrich Co., Milan, Italy.

### 2.2. Preparation of Adjuvant–Antigen Complex

Alhydrogel^®^ was diluted from the stock to the final concentration of 1 mg Al/mL. An aqueous stock solution of OVA or HSA (10 mg/mL) was prepared by dissolving the protein in distilled deionized water (milli-Q H_2_O), 14 MΩ resistance. Nanoparticles (NPs) were obtained by sonication via Vial Tweeter sonicator (UIS250v, Hielscher, Teltow, Germany) for 10 min, intensity 0.75% and pulse every 0.5 s. The sonicator deliver up to 5–10 watts to each sterile 1.5 mL vial placed in the instrument and avoids cross-contamination of samples. A series of adjuvant–antigen mass ratio was prepared (adjuvant:antigen 1:5, 1:4, 1:3, 1:2, 1:1), maintaining the protein’s concentration. This corresponds to 1 mg of alum mixed with 1 mg of the OVA protein for the 1:1 sample.

All adjuvant:antigen solutions were vigorously stirred, and complex formation was achieved in a rotating wheel for 1 h under a controlled temperature, mimicking the preparation of vaccine formulation for in vivo experiments.

### 2.3. Particles Size, Polydispersity Index

The average particle size and polydispersity index (PDI) were determined by dynamic light scattering (DLS), using a Zetasizer (Malvern Panalytical Ltd., Malvern, UK) at 25 °C, using a back-scattering angle of 173°. Measurements were performed immediately after particle dilution or preparation and each measurement was the average of 11 data sets acquired for 10 s each, with a 30 s delay between measurements. Samples were diluted 10 times to reach an attenuation factor of 7 or 8. Measurement of particle size were performed in deionized Milli-Q water, if not differently specify.

### 2.4. Surface Charge Studies: Determination of Z-Potential, Titration of Zeta-Potential against pH

Z-potential values of particles were measured by electrophoretic light scattering (ELS) using a Zetasizer ZS-Nano (Malvern Instruments, Beckman Coulter, CA, USA), at 25 °C. Measurements were performed immediately after particle dilution, and each measurement was the average of five data sets acquired with an automatic number of runs and 30 s delay between measurements, using the Smoluchowski fitting mode.

Titration curves (Z-potential vs. pH) and isoelectric point (IEP) were measured with MPT-2 accessory of Malvern Zetasizer Nano by monitoring the change in surface charge with constant and controlled pH variation of the buffer. Samples were brought to acidic pH with 0.25 M HCl solution and then titrated with the automatic addition of 0.25 M NaOH to obtain eight experimental points in the pH range 3.5 to 11.4. DTS1070 or high salt cuvette were used for measures in deionized Milli-Q water and 0.9% NaCl, respectively.

### 2.5. Particles Morphology by TEM Analysis

Particles were characterized by transmission electron microscopy (TEM). Images were obtained using a JEOL JEM 2100 microscope (JEOL, Milan, Italy) operating at 120 kV, placing a 3 µL drop of 2 µg/mL suspensions (AH and AH-NPs) on a Formvar Carbon coated 200 mesh copper grids (Agar Scientific, Stansted, UK), previously irradiated by Leica EM ACE200 (Leica Microsystems, Milan, Italy) for 30 s, 10 mA and dried in a desiccator at room temperature overnight.

### 2.6. Protein Electrophoresis

Sodium dodecyl sulphate–polyacrylamide gel electrophoresis (SDS-PAGE) was used to detect unbound protein on supernatants (SN). After 1 h of incubation in a rotating wheel, samples were centrifuged for 10 min at 10,000× *g* at room temperature and SN were collected. The same volume of SN for each sample was also loaded on Protein LabChip gel (according to manufacturer’s instruction), and the presence of unbound protein was detected with Agilent Protein 80 kit with Agilent 2100 Bioanalyzer (Agilent Technologies, Santa Clara, CA, USA). The starting amount of protein, as one of the samples, was loaded as the control. The gel contains three internal controls, which allow for the quantitation of the detected protein by Agilent 2100 software (version B.02.10SI764).

### 2.7. Release of Antigen from Adjuvant Particles

Vials containing adjuvant:antigen samples at ratio 1:2 and 1:1 were incubated at 37 °C in a thermoblock incubator, mimicking experimental in vitro conditions. A sample was withdrawn at each time point (24 and 48 h) and centrifuged 10 min at 10,000× *g* at room temperature. SN was collected and free antigen content was determined by SDS-PAGE and with the protein Bioanalyzer instrument. The percentage of antigen released was calculated considering the total protein content as 100%.

### 2.8. Circular Dichroism

Vials containing the adjuvant:antigen 1:1 sample were centrifuged 10 min, at 10,000× *g* at room temperature. SN was collected and run on SDS-PAGE gel chip to confirm the absence of unbound protein in the SN. Pellets were suspended in an equal amount of Milli-Q H_2_O and diluted 25 times. CD spectra were acquired at 25° with CD spectropolarimeter (JASCO Inc., Easton MD, MA, USA) equipped with a Peltier temperature-controlled cell holder. The adjuvant alone, at the same concentration as the one in the sample, was set as a baseline, while Milli-Q H_2_O was set as the baseline for the antigen alone. Spectra were measured in quartz cuvette with 0.5 cm optical length. Each spectrum was obtained as an average of 4 scans, from 260 to 190 nm with a 1-nm bandwidth pass. Changes in protein spectra structure of the adjuvant:antigen complex, was compared to the spectra of protein alone. Data analysis was performed using the Dicroweb web-based service [29] or Bestsel software [12]. The Dichroweb spectral deconvolution was performed with the reference dataset 4 using the CDSSTR algorithm.

## 3. Results and Discussion

### 3.1. Synthesis and Characterization of Nanoadjuvants

**Figure 1:**
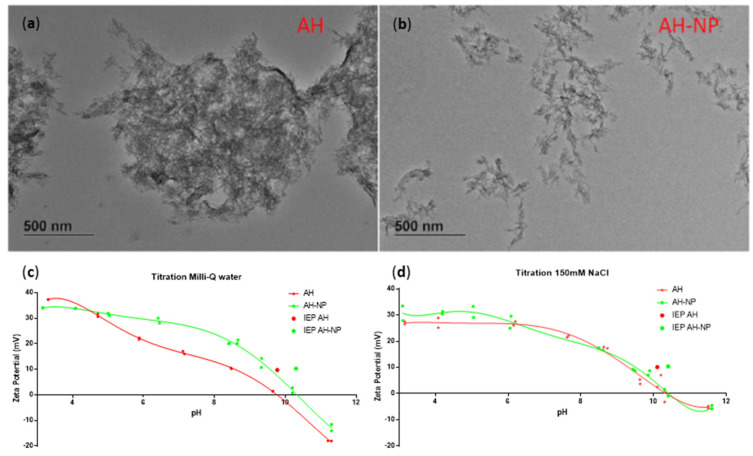
Characterization of AH and AH-NP. TEM analysis of (**a**) AH and (**b**) AH-NP. Z-potential vs. pH titration of AH, AH-NP in (**c**) Milli-Q water and (**d**) isotonic NaCl (0.9%).

Aluminum hydroxide particles (AH) were obtained by diluting Alhydrogel^®^ stock solution in Milli-Q H_2_O and vortexing at high speed for 1 min. As expected, the sample obtained was composed of aggregates of AH fibers in the size range of 1 to 10 µm. Figure 1a shows a representative electron micrograph image of such aggregates, where aluminum hydroxide fibers are clearly visible. To obtain aluminum hydroxide particles in the submicron size range we considered different preparation methods normally used in the pharmaceutical sector, such as the mechanical microfluidization method and high-power sonication. In particular, we selected the vial tweeter sonicator that allows preparing samples in closed containers, thus eliminating the risk of sample contamination (or release of titanium particles) inherent with using a probe sonicator [30]. In addition, samples obtained with the vial tweeter sonicator (Figure 1b) showed a better size homogeneity (lower polydispersity index, PDI) compared to those prepared with the microfluidifier (Supplementary Materials, Table S1).

The size of AH and AH-NP were measured both by DLS and by TEM. AH-NP have a size of 180 nm with a PDI of 0.25, while AH have a size of 850 nm and PDI of 0.19. These values are in accordance with the electron micrographs of Figure 1 that show the presence of aggregates larger than 1 μm in the case of AH, and much smaller particles for AH-NP.

The surface charge of aluminum adjuvants is a key property; in fact, its value dictates the capacity of the adjuvant to bind (by electrostatic interaction) negatively charged or positively charged protein antigens. Due to their amphoteric nature, aluminum adjuvants and protein antigens, exhibit pH-dependent charge [31]. These adjuvants can therefore change the overall surface charges based on the pH of the solution. Thus, the variation of the surface charge as a function of pH is essential to identify the best pH value where the adjuvant can bind a given antigen. Figure 1c,d shows the Z-potential of AH and AH-NP as a function of pH. AH has an isoelectric point of around 9.8, meaning that it is positively charged (more than +10 EV) at pH below 9.8 and negatively charged at pH above 10. AH-NP has a similar isoelectric point (10.3) with a slightly different shaped Z-potential-pH curve (green curve on Figure 1c). Thus, at neutral pH, AH-NPs are slightly more positively charged (+25 mV) compared to AH adjuvant (+15 mV).

The difference between AH and AH-NP curves, can be explained by the change of active surface (increased for AH-NP) and by the different proportion of titratable groups. In isotonic solution (Figure 1d), titration curves of both AH and AH-NP flatten, suggesting salification and fewer surface charged groups.

AH-NP tend to increase size and probably aggregate with time and temperature. Particles diluted in Roswell Park Memorial Institute (RPMI) 1640 culture media supplemented with 10% FBS and kept at 37 °C for 24 h showed an increase in size from 180 nm to 600 nm (Supplementary Materials, Table S2 and Figure S1). Similar increases in size were measured for AH-NP kept at 4 °C for one week. These results indicate that AH-NP has only a limited colloidal stability (as was expected from the mildly positive Z-potential) and highlight the need for using the samples immediately after preparation.

### 3.2. Synthesis of Nanoadjuvant–Antigen Complexes

We used OVA as a “classical” model antigen to develop and test the different measurements for the characterization of the adjuvant-antigen systems. The first consideration for a successful formation of aluminum adjuvant-OVA complexes is the relative charge of the two components at different pH values. As shown before, AH and AH-NP are positively charged below IEP, while OVA (with predicted IEP of 5.2) is negatively charged at pH higher than its IEP.

**Figure 2:**
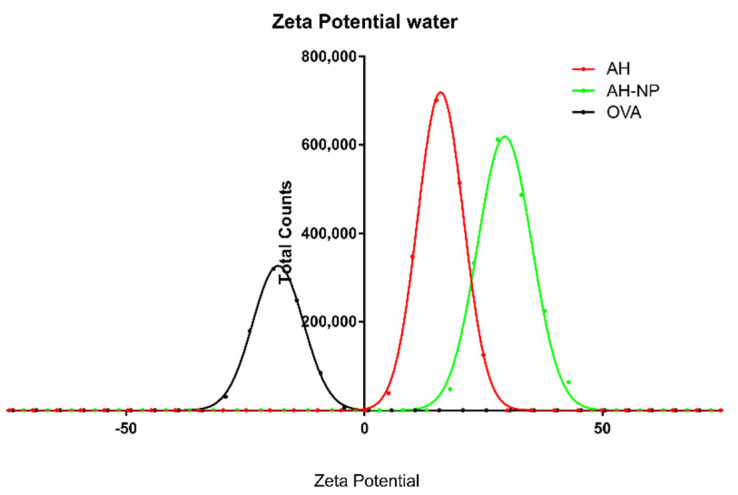
Zeta potential measurements of AH (red), AH-NP (green) and OVA (black) at pH 7.0.

The measurement of Zeta potential as a function of pH for OVA (Supplementary Materials, Figure S2) shows that (as expected) the protein is negatively charged at pH higher than 5.2. Thus, as shown in Figure 2, at neutral pH OVA is negatively charged (−30 mV), while AH and AH-NP are positively charged (+15 mV and +30 mV, respectively).

Thus, at this pH there is a strong possibility that OVA binds to aluminum adjuvants via electrostatic interactions.

### 3.3. Measuring Adjuvant Loading Capacity

The relative capacity of AH and AH-NP to bind OVA has been tested by adding increasing amounts of the aluminum adjuvants to a constant concentration of the protein. Upon incubation and formation of the adjuvant–antigen complexes, the sample was centrifuged. The pellet (containing the complex) was removed and the amount of non-bound protein in the supernatant was measured with different techniques. In particular, the use of chip-based capillary gel electrophoresis allowed us to quantify the amounts of non-bound OVA in the supernatant with a high sensitivity and accuracy.

**Figure 3:**
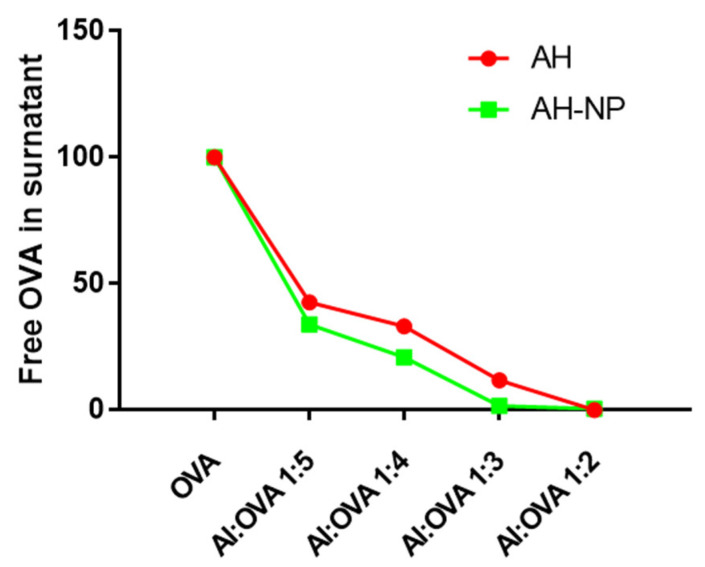
Chip-based capillary electrophoresis as % of free protein in the supernatant at variable adjuvant-OVA mass ratio for AH:OVA samples (red dots) and AH-NP:OVA (green squares). Lines connecting the experimental points are for guiding the eyes only.

Figure 3 shows the amount of free OVA protein in solution as a function of the AH:OVA and AH-NP:OVA mass ratio. Data shows that, both for AH:OVA and AH-NP:OVA, all added antigen was practically bound to the adjuvant at a mass ratio of 1:2, meaning that 1 mg of aluminum hydroxide can completely bind to 2 mg of OVA protein. A more detailed analysis indicates that AH-NP has a slightly higher capacity to bind to the OVA antigen compared to AH. Plotting aluminum content vs. the fraction of bound OVA shows that AH-NP require around 12% less aluminum to bind the same quantity of OVA compared to AH (Supplementary Materials Figure S3). Making the reasonable assumption that AH and AH-NP have similar binding strengths, this increase in loading capacity should be due to the increased surface area available on AH-NP to bind the antigen.

The results show that in the complexes AH:OVA 1:2 and AH-NP:OVA 1:2, all the added OVA antigen became stably bound to the adjuvant, they were thus selected for the subsequent in-depth characterization. The measured surface charges of the two complexes (Z-potential) of −6.4 mV for AH:OVA and −7.3 mV for AH-NP:OVA indicated that the protein molecules likely covered the whole surface of both AH and AH-NP adjuvants (Supplementary Materials, Figure S4 showing TEM and Z-potential data). In addition, both complexes showed an increase in size compared to the adjuvants alone. AH-NP:OVA significantly increased the size from 200 nm to 400 nm and the polydispersity from 0.25 to 0.4 compared to AH-NP. AH:OVA also showed large aggregates ranging from a size of 1 µm to more than 10 µm. Apart from the increase of size, the complexes were quite stable: both showed no detectable release of OVA in the supernatant after incubation for 48 h at 37 °C (Supplementary Materials, Figure S5), while the size of AH-NP:OVA increased only marginally (Z average of 420 nm) after 24 h at 37 °C.

### 3.4. Antigen Structure and Stability in Adjuvant–Antigen Complexes

The structure of the protein antigen bound to the adjuvant is a key determinant of its immunogenicity. Protein adsorbed onto a solid surface can unfold, expose cryptic epitopes, activate the inflammatory pathways [32], and their structure can vary significantly compared to the structure in solution [9].

To date, it remains challenging to obtain structural information on proteins adsorbed on solid surfaces (such as aluminum adjuvants) [33]. Here, we used CD, a well-known biophysical technique, to measure the secondary structure changes of OVA bound to both AH and AH-NP adjuvants.

**Figure 4:**
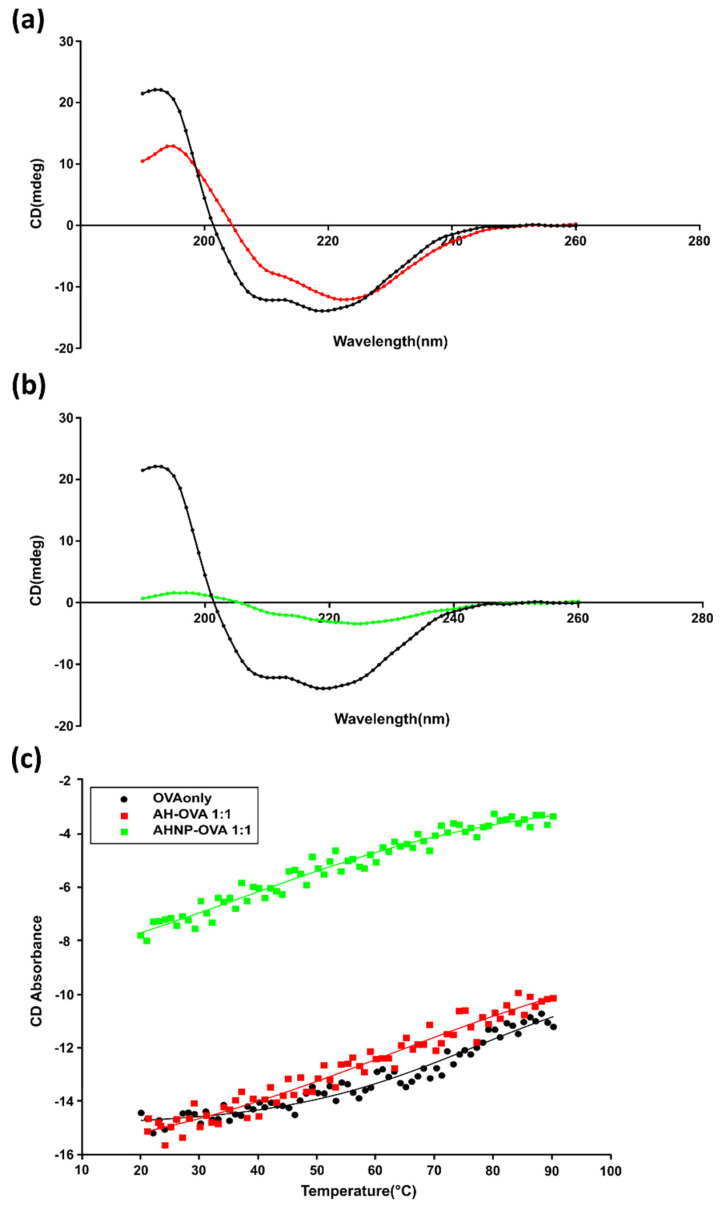
CD spectra of OVA bound to (**a**) AH or (**b**) AH-NP at 1:1 ratio. (**c**) CD thermal unfolding of free OVA, AH:OVA 1:1, AH-NP:OVA 1:1. Experimental points are shown as unconnected filled symbols (OVA: black circles; AH-OVA: red squares; AHNP-OVA: green squares). Nonlinear square fitting to Boltzman-type equation to each experimental data set as continuous lines (OVA: black; AH-OVA: red, AHNP-OVA: green).

Figure 4 shows the CD spectra in the region 190–260 nm (sensitive to the secondary structure of proteins) of free OVA (black) and of the adjuvant–antigen samples at a mass ratio of 1:1 for both AH:OVA (red) (Figure 4a) and AH-NP:OVA(green) (Figure 4b).

The OVA CD spectra changes substantially when bound to the adjuvant surface compared to the free protein in solution. Using well-established methods for deconvoluting the CD spectra [29], it is possible to estimate the secondary structure elements of the OVA protein bound to the adjuvant and to compare to the free protein in solution. Table 1 reports the amount of each secondary structure element (helix, strand, turns, unordered) for the different samples. Upon binding to AH-NP OVA, the amount of ordered structures drastically changes, including a decrease in the α-helix (from 74% to 16%), and an increase in the β-sheet and turn elements (from 14% to 33% and from 8% to 24%, respectively). OVA also significantly increased the amount of unordered structure (from 5% to 27%). The changes in OVA secondary structure after binding to AH are smaller; the most significant is a slight decrease in α-helix content (from 74% to 69%) and an increase in unordered structure (from 5% to 13%).

The changes in the structure of OVA in complex with the aluminum adjuvants is a direct consequence of the protein binding to the adjuvant’s surface. In fact, the experimental procedure requires the formation of the adjuvant:OVA complex, the separation of the non-adsorbed OVA by centrifugation, the control (by gel elecrophoresys) of the amount of free protein in the supernatant, and the CD measurement of the resuspended pellets. Our results (Supplementary Materials, Figure S5) show almost no OVA protein in the supernatant at adjuvant:antigen ratio of 1:2 and 1:1.

CD measurements of adjuvant:OVA samples allow for the determination of the changes in stability of the antigen, by measuring its thermal unfolding. Thermal unfolding was assessed by recording the intensity of the CD signal at 222 nm (characteristic of the α-helical structure) as a function of the temperature (Figure 4c). The fitting of the experimental data to a Boltzmann-type equation A + (B − A)/(1 + exp(x − x0)/dx (where x is the temperature, x0 the melting temperature, and dx the width of the thermal transition) gave a melting temperature of 77 °C for free OVA, 63 °C for AH:OVA and 38 °C for AH-NP:OVA samples.

The decrease in melting temperature for OVA adsorbed on the aluminum hydroxide adjuvants (compared to the OVA in solution) indicates a decrease in the stability of the protein bound to the adjuvant surface. These results are in good agreement with the reduction in secondary structure elements seen for OVA protein adsorbed on the adjuvant surface.

The reduction in melting temperature is much more pronounced for OVA bound to AH-NP than for OVA bound to “standard” AH.

In addition, the width of the unfolding transition (dx) shows significant changes: it increased from 17 °C for OVA in solution to 27 °C and 24 °C for AH:OVA and AH-NP:OVA, respectively. The parameter relates to the steepness of the unfolding transition and it is linked to the overall globular structure of the proteins, with the steeper unfolding being characteristic of a more globular protein folding.

The combined results of the different CD measurements (decrease in secondary structure elements, decrease in melting temperature, less steep unfolding) indicate that the OVA protein bound to the adjuvant surface has a less compact tertiary structure. This effect is much more evident for OVA bound to adjuvant nanoparticles compared to the “classical” adjuvant.

We repeated similar measurements for human serum albumin (HSA) bound to both AH and AH-NP adjuvants. HSA is the most abundant protein in human plasma and its availability made it suitable for studies as a model protein. It is a well-studied and characterized protein, and a carrier for endogenous and exogenous compounds delivery [34].

The analysis of CD data (Supplementary Materials, Figure S6, and Table S3) shows a decrease in α-helical content (from 80% to 60% and 55% for HSA bound to AH and AH-NP, respectively). The CD-detected thermal unfolding (Supplementary Materials, Figure S7) shows a decrease in melting temperature (from 76 °C for HSA in solution to 61 °C and 45 °C for AH-HSA 1:1 and AHNP-HSA 1:1, respectively) for HSA adsorbed on the adjuvant surface. In addition, there is a significantly less steep unfolding (dx from 10 °C for HSA in solution to 19 °C and 18 °C for AH-HSA 1:1 and AHNP-HSA 1:1, respectively).

These results are similar to those obtained for OVA: the HSA bound to the adjuvants has a lower secondary structure content, lower thermal unfolding, and less steep unfolding compared to HSA in solution. This indicates that also HSA bound to the adjuvants has a less compact tertiary structure and reduced stability. In addition, these effects are more pronunced in the case of AH-NP compared to the classical AH adjuvant, in a similar fashion as seen for OVA bound to AH-NP and classic AH. Previous work using scanning calorimetry measurements has shown a reduced thermal stability of the protein antigen when adsorbed onto aluminum salt [13]. This phenomenon could facilitate the presentation of the antigen, and is thus considered one of the mechanisms of adjuvanticity of aluminum adjuvants [13].

According to a recent model for the influence of protein fold stability on the availability of T- and B-cell epitopes, each protein has an optimal conformational stability for immunogenicity [9]. Protein antigens at the two extremes of fold stability would result in reduced immunogenicity: highly destabilized proteins are degraded too early, while hyperstabilized proteins undergo a too-slow proteolytic degradation, resulting in reduced antibody responses [9].

Our results show that adjuvants slightly reduce the stability for both OVA and HSA and this effect is more pronounced with AH-NP compared to AH. Overall, this indicates that the binding of proteins to aluminum adjuvants modulates the fold stability of the antigens. It is a reasonable working hypothesis that the absorption of protein antigens on aluminum adjuvant slightly reduces the conformational stability of the protein, leading to an increase in the exposure of T- and B-cell epitopes, which could contribute to the higher immunogenicity of protein-antigens systems compared to protein alone.

In addition, the method developed here can be used with any protein-bound to aluminum adjuvants, thus allowing to directly measure the conformational stability of any protein adsorbed on the adjuvant surface. This will provide an experimental technique to now assess the modulation of antigen fold stability in different vaccine formulations. For example, it will be possible to correlate the aging of some aluminum-based subunit vaccines with enhanced immune response, as in the case of the Diptheria toxoid vaccine [35].

### 3.5. Physicochemical Characterization Cascade for Nanoadjuvant–Antigen Systems

**Figure 5:**
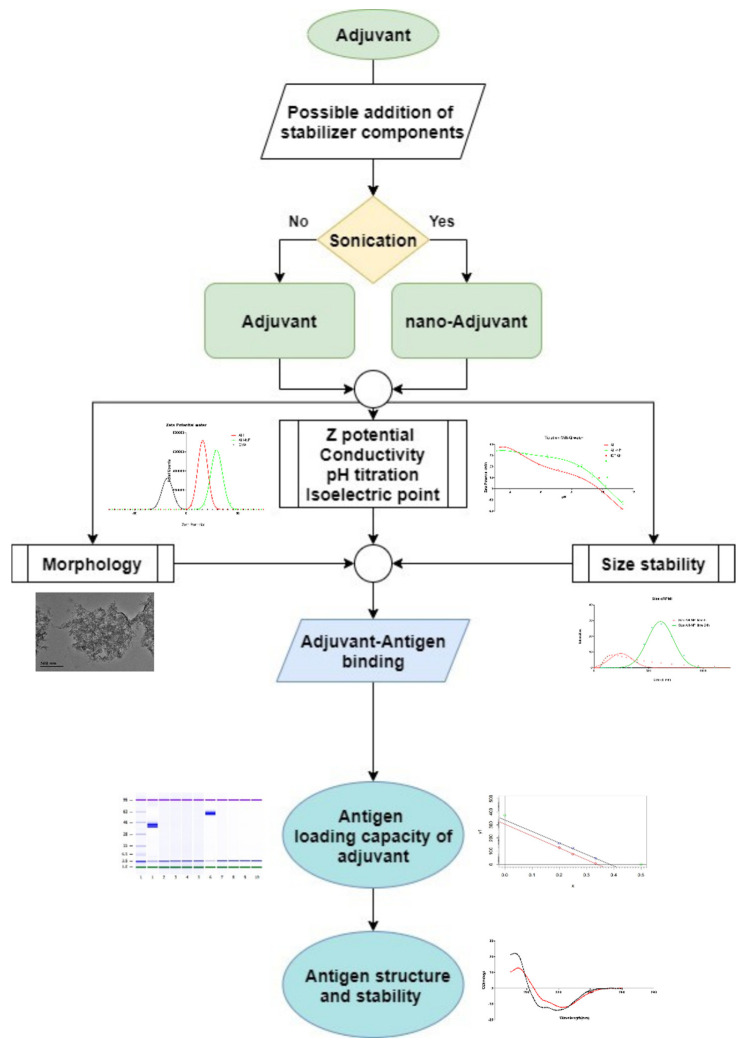
Schematic overview of the physicochemical characterization of nanoadjuvant–protein antigen systems used in this study.

Accurate characterization of physicochemical properties of adjuvants is essential for developing more effective vaccines; for example, as shown here, to identify the pH values where antigen and adjuvant maximize their interaction. Here we have shown that using a systematic approach, making use of the different existing techniques developed and applied in various scientific fields (such as nanotechnology, biotechnology, and biochemistry) it is possible to synthesize nanoadjuvant–antigen complexes and to accurately measure their key properties. Figure 5 summarized the process we developed and employed in this work. It can be used as a general, stepwise characterization cascade for an accurate physicochemical characterization of nanoadjuvant–antigen systems.

Following the identification of the most appropriate adjuvant (and eventually stabilizers), the first step consists of the preparation of the nanoadjuvant, its basic properties, average size, and electrostatic properties, which are determined. Measuring the Z-potential at different pH values allows determining its isoelectric point and estimate its colloidal stability. More in-depth analysis is needed to measure the stability in relevant physiological buffers (aggregation propensity with DLS), while the morphology of the nanoadjuvant is determined by electron microscopy.

The obtained information allows determining the best pH for the formation of the antigen–adjuvant complex. Another fundamental property is the antigen-loading capacity of the adjuvant; this can be measured with high sensitivity and accuracy by first separating the complex by centrifugation and then measuring the amount of adjuvant-bound protein by digital electrophoresis.

Finally, the structure and stability of the antigen in the antigen–adjuvant complex is analysed by CD. These measurements allow to directly quantify the changes in fold stability of the antigen adsorbed on the adjuvant surface and eventually to modulate it (by altering the formulation) to improve the immune response.

This characterization cascade has been applied to the nanoaluminum–OVA system (and confirmed using nanoaluminum–HSA), it has the advantage that can be applied to almost any combination of nanoadjuvant–antigen system.

## 4. Conclusions

The accurate measurements of the physicochemical properties of adjuvants and adjuvant–antigen systems is of critical importance for the rational design of vaccine formulation. The characterization cascade presented here follows a stepwise approach of increased complexity, starting from the nanoadjuvant alone, up to the very challenging measurement of the structure of the antigens adsorbed on the adjuvant. In particular, the use of the CD technique allows for direct measurement of the structural stability of the antigen adsorbed on the aluminum adjuvant surface. This allows for a direct and simple experimental correlation between the immunogenicity and fold stability of the adsorbed antigens in different vaccine formulations.

This characterization cascade has been developed and tested for nanoaluminum–OVA (and HSA) protein antigens but generally, it can be easily applied to any adjuvant–antigen combination. It will contribute to the development of better antigen–adjuvant complexes and immunological outcomes, ultimately leading to improved vaccines.

## Figures and Tables

**Table 1:** Secondary structure element content of free OVA, AH-NP:OVA 1:1, AH:OVA 1:1.

Sample	Helix	Strand	Turns	Unordered
Free OVA	0.74	0.14	0.08	0.05
AH-NP:OVA 1:1	0.16	0.33	0.24	0.27
AH:OVA 1:1	0.69	0.11	0.05	0.13

## Supplementary Materials

The following are available online at https://www.mdpi.com/article/10.3390/vaccines9060544/s1, Figure S1: Change in the size of AH-NP incubated in RPMI culture media, Figure S2: Measurement of Zeta potential as a function of pH for ovalbumin, Figure S3: Plot of Aluminum mass vs amount of free antigen, Figure S4: TEM micrograph of AH:OVA 1:2 complex, Figure S5: Chip-based capillary electrophoresis, Figure S6: Secondary structure of HSA bound to AH or AH-NP, Figure S7: CD thermal unfolding of free HSA, AH:HSA 1:1, AH-NP:HSA 1:1, Table S1: Comparison with different methods of aluminum nano-particles preparation, Table S2: DLS of AH-NP at time 0 and after 24 h at 37 °C, Table S3: Secondary structure elements content of free HSA, AH:HSA 1:2, AH-NP:HSA 1:2.
