# Supplementary material for: Physicochemical Characterization Cascade of Nanoadjuvant–Antigen Systems for Improving Vaccines
Source: Vaccines (Basel). 2021 May 21;9(6):544. doi: 10.3390/vaccines9060544 (PMC8224364; doi:10.3390/vaccines9060544)
Supplement: Supplementary file 1 [file vaccines-09-00544-s001.zip › vaccines-1198443-supplementary.pdf]

Physico-chemical characterization cascade of (nano)adjuvant-antigen systems for improving vaccines

Giuditta Guerrini[1], Antonio Vivi[1], Sabrina Gioria[2], Jessica Ponti[2], Davide Magrì[2], Arnd Hoeveler[2], Donata Medaglini[1], Luigi Calzolai[2]*

[1]Università di Siena, Dipartimento di Biotecnologie Mediche LAMMB (Biotechnology and Molecular Microbiology Lab)

[2]European Commission, Joint Research Centre (JRC), Ispra, Italy

Sample-name	Sample-number	Instrument	Processing	Z-average (nm)	PDI
AH 1:10	1	vial twitter	5 min sonic.	309	0,50
AH 1:10	2	vial twitter	10 min sonic.	180	0,25
AH 1:10	3	vial twitter	20 min sonic.	248	0,43
Sample 2	2.1	Microfluidizer M110P	10 pass 30 kpsi	163	0,48
Sample 2	2.2	microfluidizer M110P	20 pass 30 kpsi	537	0,56

Table S1. Comparison with different methods of aluminum nano-particles preparation

Synthesis of nano aluminum hydroxide particles using two different methods: vial twitter sonicator (Hielscher Ultrasound Technology) for samples 1, 2, and 3 or with high shear fluid processing (Microfluidizer M110P, Microfluidics International Corporation) for samples 2.1 and 2.2. Processing parameters (time for sonication and number of passages plus pressure) are reported for the different samples. Average size and polydispersity index have been measured with DLS (Malvern ZS).

Time	0		24h at 37°C	
	**Z-average**	**PDI**	**Z-average**	**PDI**
AH-NP	182	0,27	597	0.08

Table S2. DLS of AH-NP at time 0 and after 24h at 37°C.

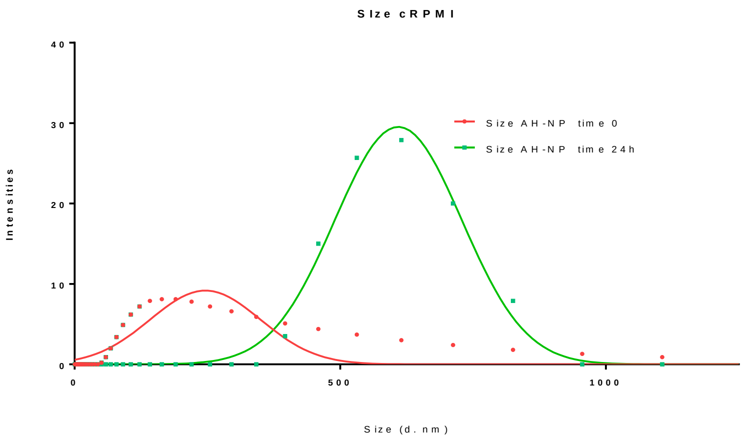

Figure S1 supporting info. Change in the size of AH-NP incubated in RPMI culture media, plus 10% FBS. After 24 hours at 37°C the particles show an increase in size from 180 nm to 600 nm

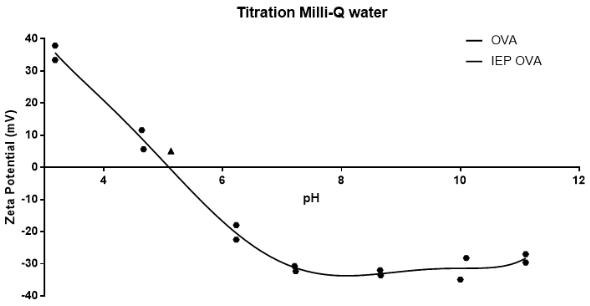

Figure S2: Measurement of Zeta potential as a function of pH for ovalbumin. Black circles: experimental points. Black triangle: OVA isoelectric point.

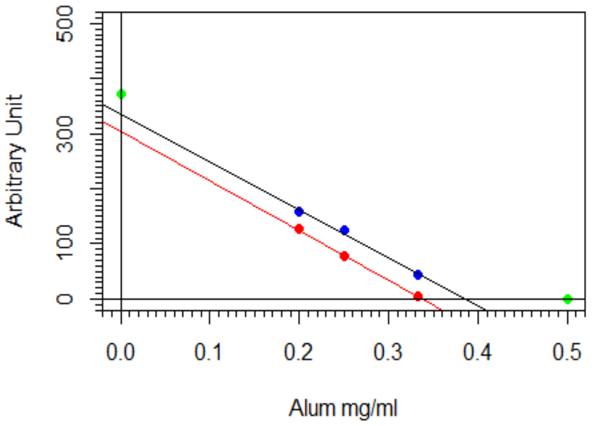

Figure S3: Plot of Aluminium mass vs amount of free antigen (arbitrary units) for AH (blue dots: experimental points, blue line: best linear fitting) and AH-NP (red dots: experimental points, red line: best linear fitting). Using the fitting results it can be estimated that all the antigen is bound when aluminium concentration is 0.385 mg/mL for AH and 0.338 mg/mL for AH-NP. This indicates that AH-NP requires around 12% less aluminium compared to AH to bind the same amount of OVA antigen.

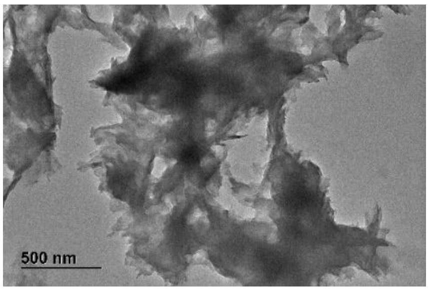

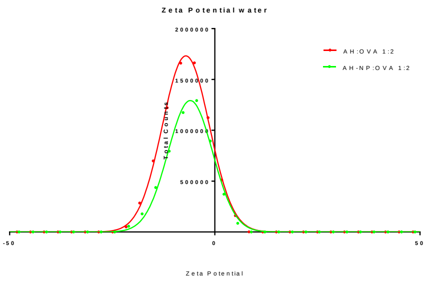

(a) **(b)**

Figure S4: (**a**) TEM micrograph of AH:OVA 1:2 complex. (**b**) Z-potential of AH:OVA 1:2 (red dots and line) and AH-NP:OVA 1:2 (green dots and line) complexes.

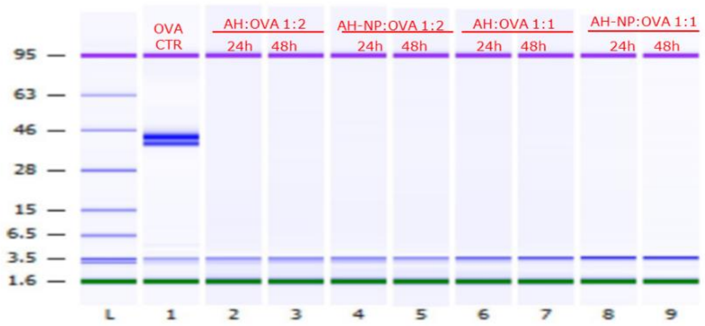

Figure S5: Chip-based capillary electrophoresis of surnatant of AH:OVA (1:2, 1:1) and AH-NP:OVA (1:2, 1:1) complex after 24h and 48h at 37°C.

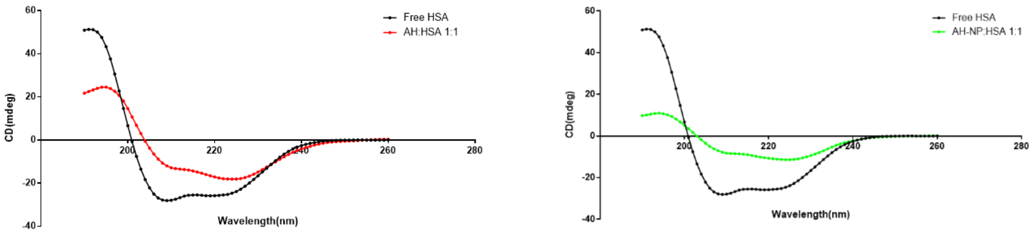

Figure S6: secondary structure of HSA bound to AH or AH-NP

Sample	Helix	Strand	Turns	Unordered
HSA Alone	0,8	0,07	0,02	0,1
AH HSA 1:2	0,6	0,13	0,08	0,19
AH-NP HSA 1:2	0,55	0,19	0,06	0,2

Table S3: Secondary structure elements content of free HSA, AH:HSA 1:2, AH-NP:HSA 1:2

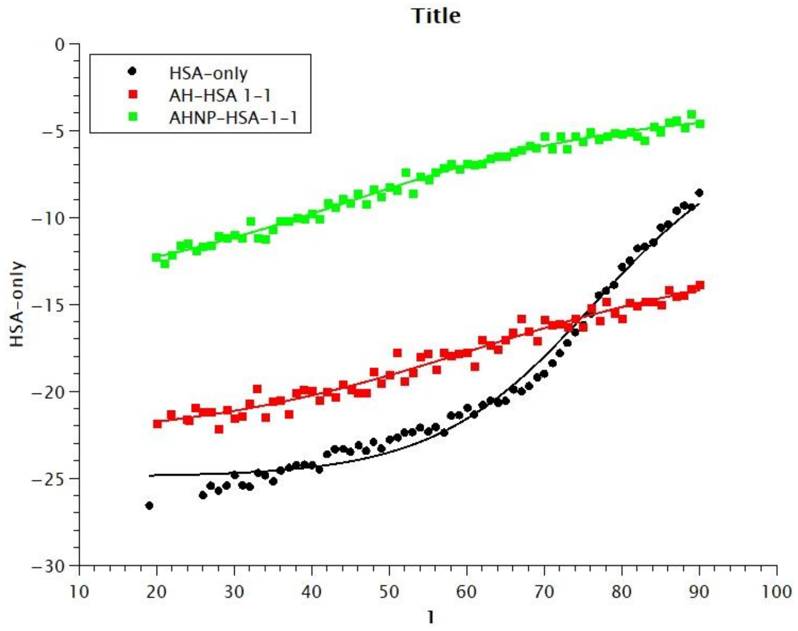

Figure S7: CD thermal unfolding of free HSA, AH:HSA 1:1, AH-NP:HSA 1:1. Experimental points shown as unconnected filled symbols (HSA black circles; AH:HSA red squares; AH-NP:HSA green squares). Non linear square fitting to Boltzman-type equation to each experimental data set as continous lines (HSA black; AH:HSA red, AH-NP:HSA green).
